# Effect of neonatal melatonin administration on behavioral and brain electrophysiological and redox imbalance in rats

**DOI:** 10.3389/fnins.2023.1269609

**Published:** 2023-10-12

**Authors:** Amanda de Oliveira Araújo, Maria Luísa Figueira-de-Oliveira, Arthur Gabriel Alves Furtado de Carvalho Noya, Vitor Palmares Oliveira e Silva, Jennyfer Martins de Carvalho, Leucio Duarte Vieira Filho, Rubem Carlos Araújo Guedes

**Affiliations:** ^1^Department of Physiology and Pharmacology, Universidade Federal de Pernambuco, Recife, Pernambuco, Brazil; ^2^Department of Nutrition, Universidade Federal de Pernambuco, Recife, Pernambuco, Brazil

**Keywords:** melatonin, brain development, anxiety, memory, spreading depression, redox imbalance

## Abstract

**Introduction:**

Melatonin (MLT) reportedly has beneficial effects in neurological disorders involving brain excitability (e.g., Epilepsy and Migraine) and behavioral patterns (e.g., Anxiety and Depression). This study was performed to investigate, in the developing rat brain, the effect of early-in-life administration of two different doses of exogenous MLT on behavioral (anxiety and memory) and electrophysiological (CSD analysis) aspects of brain function. Additionally, brain levels of malondialdehyde (MDA) and superoxide dismutase (SOD), both cellular indicators of redox balance status, were evaluated. We hypothesize that MLT differentially affects the behavioral and CSD parameters as a function of the MLT dose.

**Materials and methods:**

Male Wistar rats received, from the 7th to the 27th postnatal day (PND), on alternate days, vehicle solution, or 10 mg/kg/or 40 mg/kg MLT (MLT-10 and MLT-40 groups), or no treatment (intact group). To perform behavioral and cognition analysis, from PND30 to PND32, they were tested in the open field apparatus, first for anxiety (PND30) and then for object recognition memory tasks: spatial position recognition (PND31) and shape recognition (PND32). On PND34, they were tested in the elevated plus maze. From PND36 to 42, the excitability-related phenomenon known as cortical spreading depression (CSD) was recorded, and its features were analyzed.

**Results:**

Treatment with MLT did not change the animals’ body weight or blood glucose levels. The MLT-10 treatment, but not the MLT-40 treatment, was associated with behaviors that suggest less anxiety and improved memory. MLT-10 and MLT-40 treatments, respectively, decelerated and accelerated CSD propagation (speed of 2.86 ± 0.14 mm/min and 3.96 ± 0.16 mm/min), compared with the control groups (3.3 ± 0.10 mm/min and 3.25 ± 0.11 mm/min, for the intact and vehicle groups, respectively; *p* < 0.01). Cerebral cortex levels of malondialdehyde and superoxide dismutase were, respectively, lower and higher in the MLT-10 group but not in the MLT40 group.

**Conclusion:**

Our findings suggest that MLT intraperitoneal administration during brain development may differentially act as an antioxidant agent when administered at a low dose but not at a high dose, according to behavioral, electrophysiological, and biochemical parameters.

## 1. Introduction

Melatonin (MLT) is an antioxidant hormone (Poeggeler et al., 1993; Boutin et al., 2023) that, when used in physiological doses, could benefit patients suffering from several diseases (Arendt, 2005), including excitability-related disorders, such as epilepsy (Gunata et al., 2020) and neonatal hypoxia-ischemia (Flinn et al., 2020; for a review, see Cardinali, 2019). The amphipathic nature of MLT facilitates its passage through the blood–brain barrier and enables its effect on the nervous system, either independent of or mediated by MLT receptors. MLT is a powerful antioxidant, free radical scavenger, and immune system regulator with an anti-inflammatory and circadian rhythm regulator role (Gunata et al., 2020). This hormone neutralizes reactive oxygen species (ROS) and enzymatically converts them into less harmful species (Reiter et al., 2017, 2018). MLT receptors have been classified into three types (MT1, MT2, and MT3). In the mammalian brain, MT1 receptors are mainly found in the suprachiasmatic nucleus, hippocampus, habenula, pituitary gland, raphe nucleus, substantia nigra and superior colliculus; MT2 receptors are located in the retina, hypothalamus, hippocampus, periaqueductal gray, thalamic and supraoptic nuclei, substantia nigra and inferior colliculus (Dubocovich et al., 1998; Lacoste et al., 2015). These two types of receptors are substantially involved in circadian rhythm regulation. The MT3 receptors are the co-substrate of the enzyme quinone reductase-2, with a putative involvement in the brain redox balance (Nosjean et al., 2001; Tan et al., 2007; Wang et al., 2019a). Furthermore, MLT levels are low in patients with neurological diseases, such as epilepsy, a brain excitability disorder that mainly affects age extremes: childhood and elderly (Sanchez-Barcelo et al., 2017). Children with drug-resistant epilepsy have more sleep disorders than healthy children (Proost et al., 2022). Besides, MLT may protect epilepsy through its antioxidant, anti-excitotoxic, and central nervous system free radical scavenging properties (Akyuz et al., 2021).

In addition to its fundamental role in circadian rhythm regulation and scavenging free radicals, MLT can improve neurobehavioral deficits (Rodriguez et al., 2004). MLT can interact with other physiological systems to control anxiety and depression (Bouslama et al., 2007; Lamtai et al., 2021). Another benefit of MLT is its influence on memory formation in the hippocampus (El-Sherif et al., 2003; Iwashita et al., 2021). MLT has also been shown to be protective against neuronal damage in the hippocampus, resulting in improved learning and memory due to its potent antioxidant properties (Soleimani et al., 2017). However, it is not yet known whether its administration in high doses would have the opposite (prooxidant) effect on behavioral and electrophysiological aspects of brain function, as demonstrated in the rat brain for other antioxidant molecules, including ascorbic acid (Mendes-da-Silva et al., 2014) and pyridoxine (Gondim-Silva et al., 2021) in an electrophysiological phenomenon known as cortical spreading depression (CSD).

CSD is a fully reversible, depolarizing “wave” of reduction of the amplitude of the electrocorticographic activity (ECoG depression), which is elicited in response to the stimulation of a point of the cortical tissue (Leão, 1944). During the ECoG depression, a direct current (DC) slow potential shift appears (Leão, 1947). This DC signal has all-or-none features, is the CSD hallmark, and is very useful in calculating the CSD velocity of propagation. Regarding the human species, CSD appears to be involved in several neurological diseases, such as migraine, epilepsy, subarachnoid hemorrhage, and traumatic brain injury (for an overview, see Lauritzen and Strong, 2017; Guedes and Abadie-Guedes, 2019). The evidence suggests that analysis of CSD propagation velocity represents a valuable index to understanding excitability-dependent aspects of brain functioning (Guedes et al., 2017). CSD has been employed in our laboratory over a few decades, and it has been largely and compellingly demonstrated that factors that affect CSD can also affect other aspects of brain function, including behavior and redox balance (for an overview, see Guedes, 2011; Guedes and Abadie-Guedes, 2019).

From the above, the present study was performed to investigate, in the developing rat brain, the effect of early-in-life administration of two different doses of exogenous MLT on behavioral (anxiety and memory) and electrophysiological (CSD analysis) aspects of brain function, as will be described below. Additionally, brain levels of malondialdehyde (MDA) and superoxide dismutase (SOD), both cellular indicators of redox balance status, were evaluated. We hypothesize that MLT differentially affects the behavioral and CSD parameters as a function of the MLT dose.

## 2. Materials and methods

### 2.1. Animals

All experimental procedures were approved by our University’s Animal Research Ethics Committee (approval protocol no. 0006/2020), whose standards comply with those established by the National Institutes of Health Guide for Care and Use of Laboratory Animals (Bethesda, MD, United States). Newborn male and female Wistar rats were suckled in a litter with eight pups. After weaning, pups were separated by sex and housed in polypropylene cages (51 cm × 35.5 cm × 18.5 cm) under controlled temperature (23°C ± 1°C) with a 12–12-h light–dark cycle (lights on at 7 p.m.). Only male pups (*n* = 40; 10 rats per group) were used in this study. Each group was formed with pups from 3 to 5 distinct litters.

### 2.2. Treatment with MLT

MLT (purchased from Sigma Aldrich, St Louis, United States) was administered every other day, from PND7 to PND27, at 2 p.m. (11 days of MLT administration). Two groups of rats (*n* = 10 per group) received intraperitoneal injections of 10 or 40 mg/kg MLT (MLT10 and MLT40 groups, respectively). MLT was dissolved in 0.1 mL of saline solution containing 5% ethanol. These experimental groups were compared with two control groups, treated with the vehicle used to dissolve MLT (vehicle group; *n* = 10) or not treated (intact group; *n* = 10).

### 2.3. Body weight

Body weight was measured using a precision digital scale (Marte, São Paulo) on PND7, PND14, PND21, and PND28.

### 2.4. Glycemia analysis

At two time points on PND27 (4 h before and 4 h after the administration of MLT), plasma glucose concentrations were determined in one-drop blood samples from the animal’s tail, using a glucometer (Accu-Chek Active, Roche, São Paulo, Brazil).

### 2.5. Behavioral tests

All behavioral tests were performed between PND30 and PND34 in the following temporal sequence: first, on PND30-32, the animals were tested in the open field apparatus (OF) for anxiety (PND30) and for two recognition memory tasks: spatial position recognition (PR; PND31) and shape recognition (SR; PND32). Second, after 48 h, the animals were tested on PND34 in the elevated plus-maze (EPM). A video camera recorded the animal’s behavioral activity in all tests, which was further analyzed with ANYmaze™ software version 4.99 m (Stoelting Co., Wood Dale, IL, United States). After each test, the device was cleaned with a 70:30 ethanol:water solution. The animals were randomly tested between 8 a.m. and 1 p.m.

The tests were conducted in a room with sound attenuation and low light intensity (red light). Before each test, the animal was introduced to the test room for 20 min to adapt to the environment (adaptation period). Each anxiety behavior test (OF and EPM) consisted of one 5-min session. In comparison, each recognition memory test (PR and SR) consisted of two 5-min sessions (the training session and test session), separated by a 40-min interval.

#### 2.5.1. Behavior indicative of anxiety: tests in the OF and EPM

The OF and EPM aim to assess the ability of the animal to recognize anxiogenic sites (the central part of the OF and the open arms in the EPM), where less time is usually spent during the test (Calza et al., 2010). The OF consisted of a circular arena made of varnished wood, with a diameter of 89 cm, surrounded by a wall, also made of wood, 52 cm high. For the OF test (PND30), the animal’s behavior was evaluated for 5 min after the adaptation period. The center of the OF was defined as an area with a diameter of 62 cm. The following four parameters were measured on the OF: (1) number of entries into the central area, (2) time spent in the central area, (3) distance traveled, and (4) immobility time. The animal’s entrance to the center was considered when its four paws were in the center. Then, memory tests of recognition of a new spatial position (PND31) and a new shape (PND32) of objects placed in the OF were performed (see Part 4.5.2 below). After 48 h, the animals were tested in the EPM (PND34).

The EPM (raised 55 cm from the floor) was made of varnished wood, consisting of two open and two closed arms perpendicular to the open arms (each measuring 49 cm × 10 cm) and connected by a 10 cm-long central square. After the adaptation period, the animal was placed in the central square facing an open arm. The following four parameters were evaluated in the EPM: (1) number of entries into the open arms, (2) time spent in the open arms, (3) distance traveled, and (4) immobility time. The animal’s entrance to one arm was considered when its four paws were in that arm.

#### 2.5.2. Behavior indicative of memory: object recognition tests

These tests assessed the animal’s ability to recognize a specific object’s novel shape or spatial position. It is based on the animal’s natural tendency to spend more time exploring a new object or a novel spatial position of a familiar object that has been previously explored (Francisco and Guedes, 2015). Ennaceur and Delacour (1988) and Dere et al. (2005) described these methods for object recognition tests, which are briefly summarized below:

(1) For the PR task, the two chosen objects were identical, made of the same material (glass), with similar interaction possibilities. Initially, the two identical objects (A and B) were placed in the OF, and the animal explored them for 5 min. After a 40-min intermission, the animal returned to the OF (second session) in the presence of the same objects (A and B); however, in this second moment, object B was located at a novel spatial position. When the animal identified the novel position, it explored the object more in the novel placement point.

(2) For shape recognition, objects had distinct geometries and different shapes, heights, and colors, which the animal could quickly identify. This condition is believed to minimize the influence of natural preferences (Viana et al., 2013). Two identical objects (A and B) were initially positioned in the arena for a 5-min exploration. After a 40-min interval, the animals returned to the arena. For this second 5-min session, the spatial position of the objects remained the same as in the first session, but object B was replaced by a novel object (C) with a different shape. The animal demonstrated that it could differentiate the shapes when it dedicated more time to exploring the novel object in this second session. To better evaluate the memory effects, the discrimination index (DI; Viana et al., 2013) was calculated using the formula DI = (TN–TF)/(TN + TF), where TN and TF represent the exploration time of the object with a novel and familiar characteristic, respectively (either by shape or spatial position). The criterion for defining exploration was based on ‘active operation,’ when the animal touched objects at least with its nose.

### 2.6. Recording of cortical spreading depression

**Figure 1:**
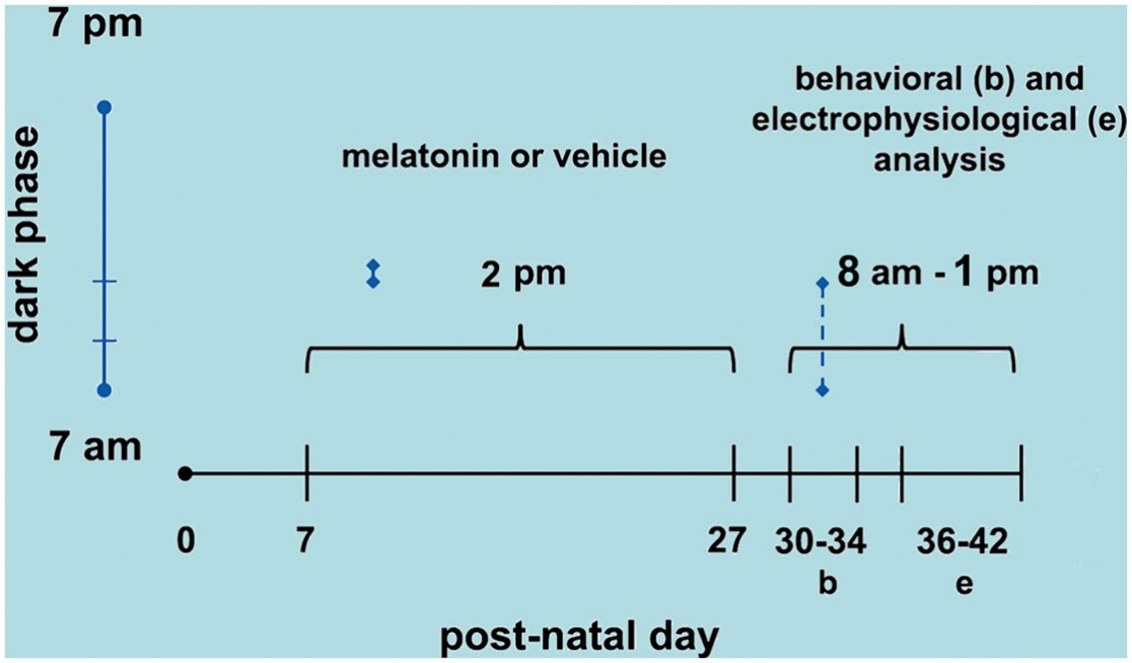
Schematic diagram showing the time points of melatonin treatment (postnatal day 7 to 27), behavioral tests (PND 30–34), and CSD electrophysiological recordings (PND 36–42).

On PND36-42, the animals were anesthetized with an intraperitoneal injection of a mixture of urethane and chloralose (1 g/kg and 40 mg/kg, respectively). Three trephine holes were drilled on the right side of the skull, aligned in the frontal-occipital direction and parallel to the midline. One hole (2–3 mm in diameter) was positioned on the frontal bone and was used to apply the cortical spreading depression (CSD)-eliciting stimulus (KCl solution). The parietal bone’s other two holes (3–4 mm in diameter) were used to record the propagated CSD wave. During surgery and recording, the animals breathed spontaneously, and their rectal temperature was monitored and kept at 37 ± 1°C. As a rule, topical application of 2% KCl (approximately 270 mM) for 1 min at a point on the exposed frontal cortical surface elicited a single episode of CSD. KCl application was repeated every 20 min during the 4-h recording session. The depression of the ECoG waves and the slow potential shift of the CSD were recorded using two Ag-AgCl-Agar-Ringer electrodes located at the parietal region on the stimulated hemisphere. A third electrode of the same type was placed on the nasal bones and was a standard reference for the other two recording electrodes. The CSD propagation velocity was calculated from the time required for a CSD wave to cross the distance between the two cortical recording points. The starting point of each ascending phase of the negative component of the slow potential shift of CSD was used as a reference point to calculate the propagation velocities of the phenomenon. Additionally, the amplitude and duration of the negative component of CSD were calculated, as previously reported (Mendes-da-Silva et al., 2014). Figure 1 shows the time points of melatonin treatment (postnatal days 7 to 27), behavioral tests (PND 30–34), and CSD electrophysiological recordings (PND 36–42).

### 2.7. Analyses of brain MDA and SOD

In the four experimental groups, malondialdehyde (MDA) and superoxide dismutase (SOD) levels were measured in 20 rats (5 animals from each group). After the CSD recording session, these 20 animals were decapitated, and the entire cortex of the right hemisphere was dissected and individually homogenated. We measured MDA levels using a thiobarbituric acid-reactive substances-based method as a way to estimate the levels of lipid peroxidation in the brain (Ohkawa et al., 1979; Mendes-da-Silva et al., 2014). Initially, 40 μL of 8.1% sodium dodecyl sulfate, 300 μL of 20% acetic acid (pH 3.5), and 300 μL of 0.8% thiobarbituric acid solutions were added to a 100-μL homogenate aliquot in a boiling water bath for 30 min. Then, the tubes were cooled with tap water, and 300 μL of n-butanol was added to the sample. After centrifuging the tubes at 2,500 ×g for 10 min, the organic phase was read at 532 nm using a plate reader. MDA values were expressed as nmol/mg of protein in the homogenate.

The levels of SOD were assessed as described elsewhere (Misra and Fridovich, 1972), based upon the brain tissue’s ability to reduce the formation of the pink chromophore, adrenochrome, from the oxidation of epinephrine. Briefly, brain homogenates were prepared at 10 mg of protein/mL dilution and added to a 96-well plate to determine enzymatic activity. Then, the solution was supplemented with 1.5 mM epinephrine. The rate of adrenochrome formation was estimated by measuring absorbance at 15-s intervals for 2 min. SOD values were expressed as U SOD.min^−1^/mg of protein, where one unit of SOD is defined as the amount of enzyme required to halve the spontaneous rate of adrenochrome formation.

The total protein concentrations in the homogenates were determined with the BCA Protein Assay Kit (Pierce, Rockford, IL, United States). Measurements were carried out in triplicate.

### 2.8. Statistics

The results are expressed as the means ± standard deviations. The four treatment groups’ ponderal, behavioral, CSD, and biochemical parameters were compared using one-way ANOVA, followed by a *post hoc* test (Holm–Sidak), where indicated. Weight differences over distinct ages were analyzed with ANOVA for repeated measures. The same animal’s blood glucose levels before and after MLT were compared using the paired *t*-test. Intergroup glucose levels were analyzed with one-way ANOVA. A value of *p* < 0.05 was considered significant.

## 3. Results

### 3.1. Body weights

**Figure 2:**
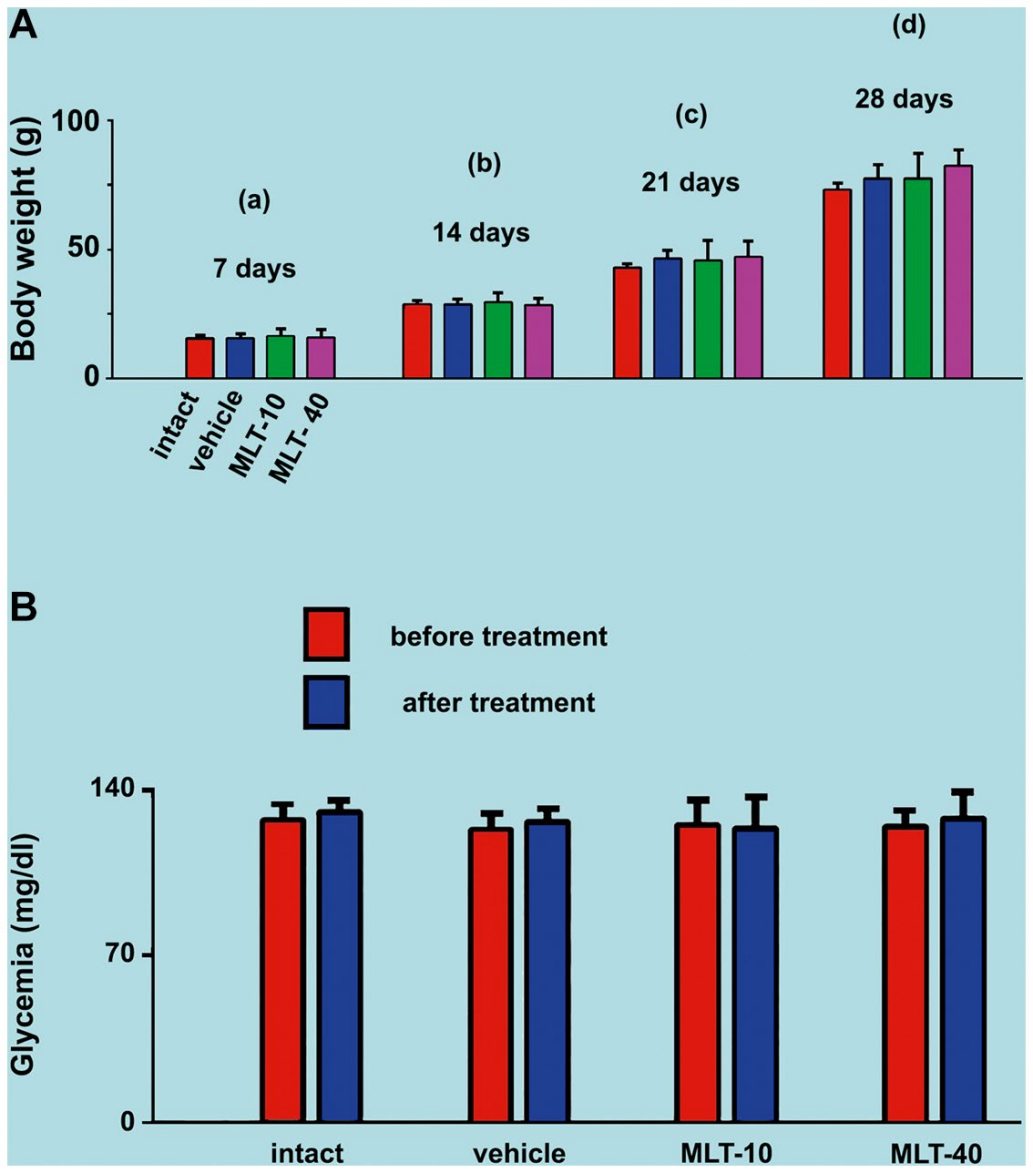
**(A)** Mean body weights of the two control groups (intact and vehicle) and those treated with MLT (10 and 40 mg/kg/day; MLT-10 and MLT-40 groups, respectively). Animals were weighed on Days 7 (a), 14 (b), 21 (c), and 28 (d) of postnatal life. MLT treatment did not influence weight gain. The two-way ANOVA for repeated measures and one-way ANOVA confirmed that, at an older age, animals from the same group were significantly heavier than at a younger age, and the absence of intergroup differences indicated a similar weight gain for all groups. **(B)** Blood glucose levels of the four groups in this study. Blood glucose was measured twice at PND28, 4 h before and 4 h after MLT administration; under these conditions, the treatment with MLT did not influence the glycemia of the animals (intragroup comparison: *p* > 0.05; paired *t*-test). The comparison between MLT-treated and non-treated groups revealed no intergroup difference [one-way ANOVA; *F*(3, 36) = 0.216; *p* = 0.885]. Data are expressed as the mean ± standard deviation. The n is 10 for each group, as stated in the methods.

As illustrated in Figure 2A, ANOVA showed no significant intergroup difference in body weight at 7 days of age [*F*(3, 36) = 0.199; *p* = 0.896], at 14 days [*F*(3, 36) = 0.352; *p* = 0.788], at 21 days [*F*(3, 36) = 0.762; *p* = 0.525], and 28 days [*F*(3, 36) = 1.939; *p* = 0.151]. The administration of the two different doses of MLT did not change the weight gain of the animals.

### 3.2. Glycemia

ANOVA revealed no intergroup difference in the blood glucose levels [*F*(3, 36) = 0.216; *p* = 0.885], i.e., at PND28 (end of MLT treatment), control groups and MLT-treated groups displayed similar blood glucose levels. MLT also had no acute effect when comparing, in the same animal, blood glucose before and after MLT administration in the two treated groups (*p* > 0.05; paired *t*-test). Data on glycemia are shown in Figure 2B.

### 3.3. Behavioral reactions

#### 3.3.1. Behavior suggestive of anxiety

**Figure 3:**
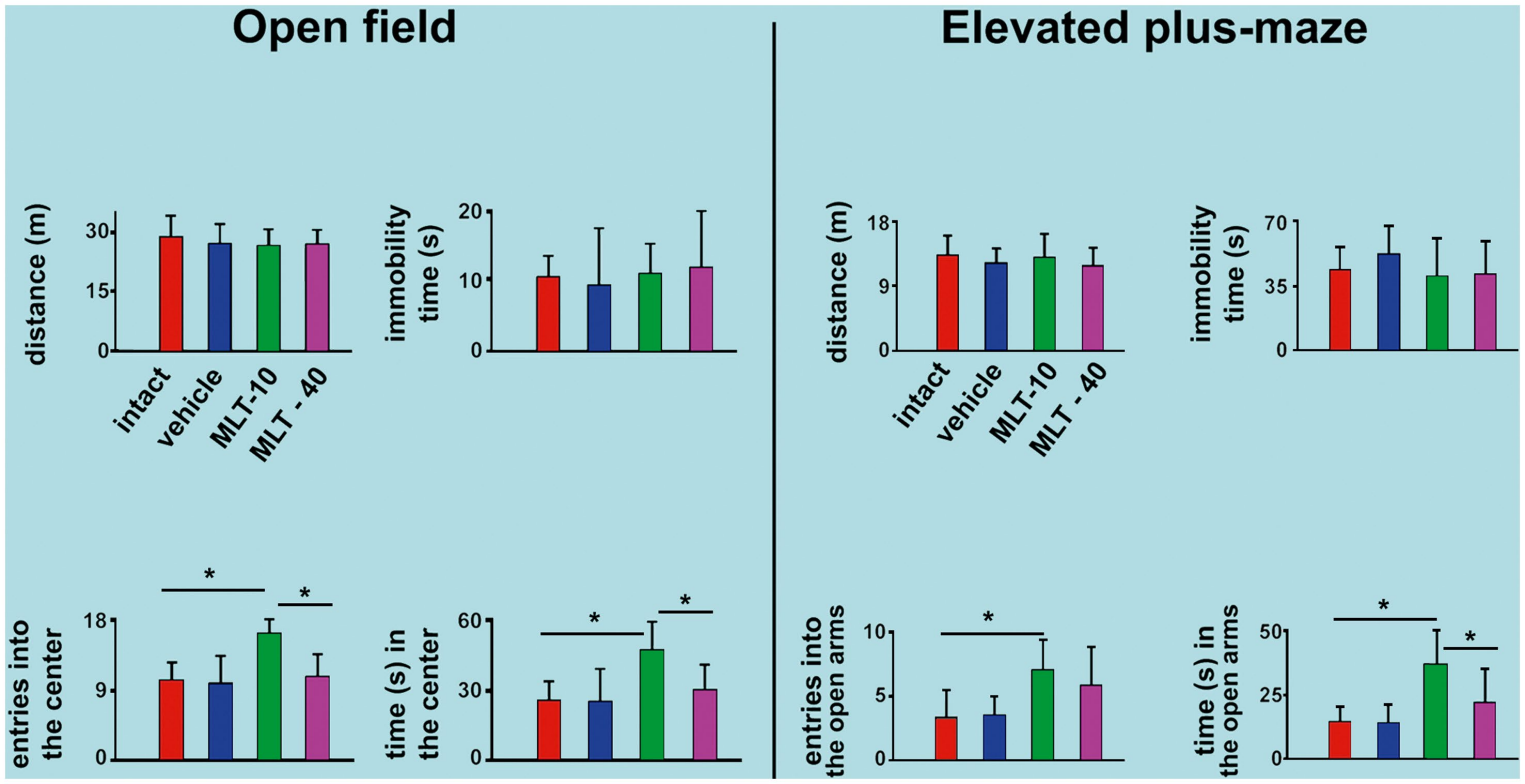
Behavioral parameters were evaluated in the open-field (OF) apparatus and the elevated plus maze (EPM) task in 30-and 34-day-old rats. In both tests, data are presented as the mean ± standard deviation. The *n* is 10 for each group, as stated in the methods. ^*^p < 0.05 (ANOVA plus Holm–Sidak test).

The effect of MLT administration on behavioral activity in the OF test is shown in Figure 3, left part. ANOVA indicated intergroup differences for the number of entries into the central area [*F*(3, 36) = 8.706; *p* < 0.001] and time in the central area [*F*(3, 36) = 5.993; *p* = 0.003]. The *post hoc* test indicated that the animals from the MLT-10 group entered the central area more often and remained there longer than the other three groups. There were no intergroup differences regarding the distance traveled [*F*(3, 36) = 0.456; *p* = 0.600] or immobility time [*F*(3, 36) = 0.194; *p* = 0.899].

The EPM test outcome is shown in Figure 3, right part. ANOVA indicated intergroup differences for the entries into the open arms [*F*(3, 35) = 4.200; *p* = 0.015] and time spent in the open arms [*F*(3, 35) = 10.036; *p* < 0.001]. The *post hoc* test (Holm–Sidak test) indicated that the MLT-10 group entered the open arms more frequently and stayed longer in the open arms than the control groups (intact and vehicle). There was no intergroup difference regarding the traveled distance [*F*(3, 35) = 0.631; *p* = 0.600] or immobility time [*F*(3, 35) = 0.852; *p* = 0.475].

#### 3.3.2. Behavior in the object recognition memory test

**Figure 4:**
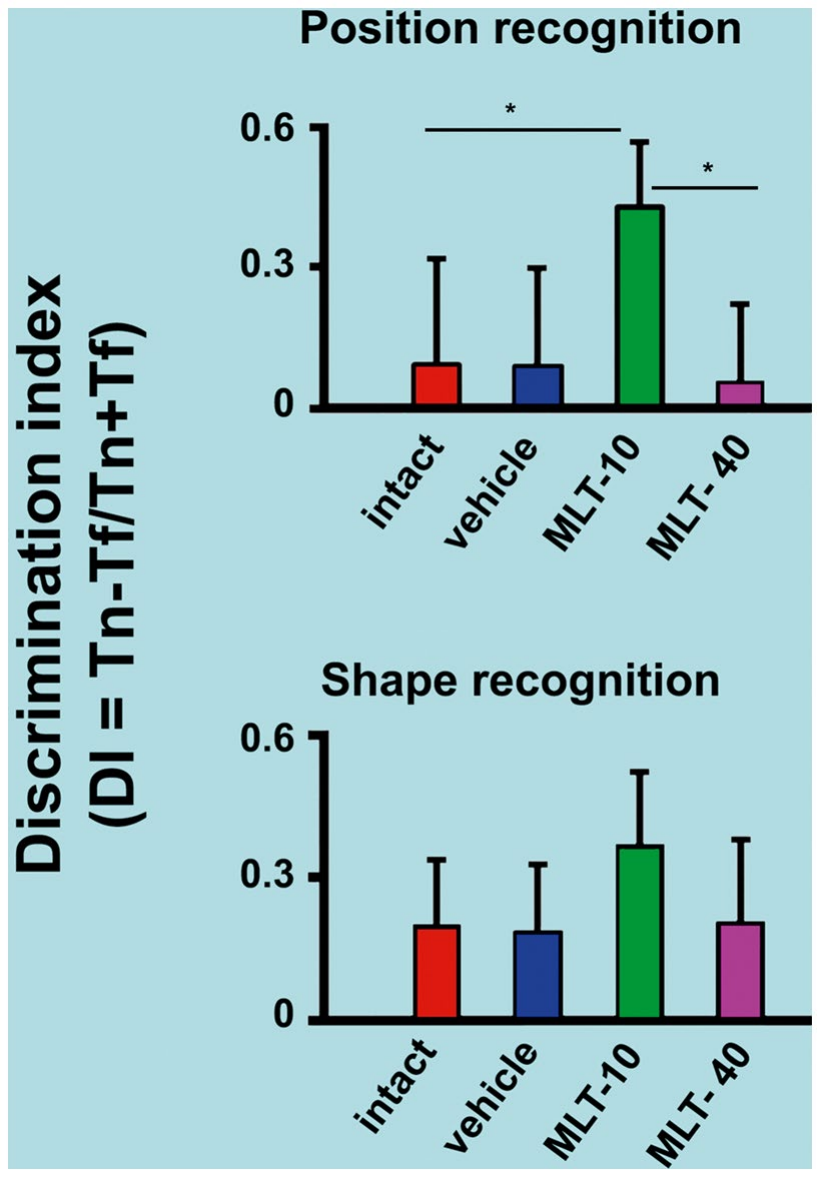
Discrimination indices of rats that were subjected to the object recognition memory test. The upper graph of this figure refers to the recognition of a new spatial position (PND31) of an already known (or familiar) object. The bottom graph represents the recognition of an object with a new shape (PND32) but in a familiar spatial position. ^*^p < 0.05 (ANOVA followed by the Holm–Sidak test). The *n* is 10 for each group, as stated in the methods.

The outcome of the object recognition memory tests (performed on PND31 and PND32) is shown in Figure 4. The upper graph refers to the discrimination indices that indicate the recognition of a novel spatial position of a familiar object. The lower graph corresponds to the discrimination indices for recognizing an object with a novel shape different from a familiar object’s. ANOVA revealed intergroup differences in spatial recognition [*F*(3, 36) = 7.813; *p* < 0.001] but not in shape recognition [*F*(3, 35) = 2.025; *p* = 0.136]. The *post hoc* (Holm–Sidak) test revealed that the MLT-10 group performed significantly better (*p* < 0.05) than the other three groups.

### 3.4. Cortical spreading depression features

**Figure 5:**
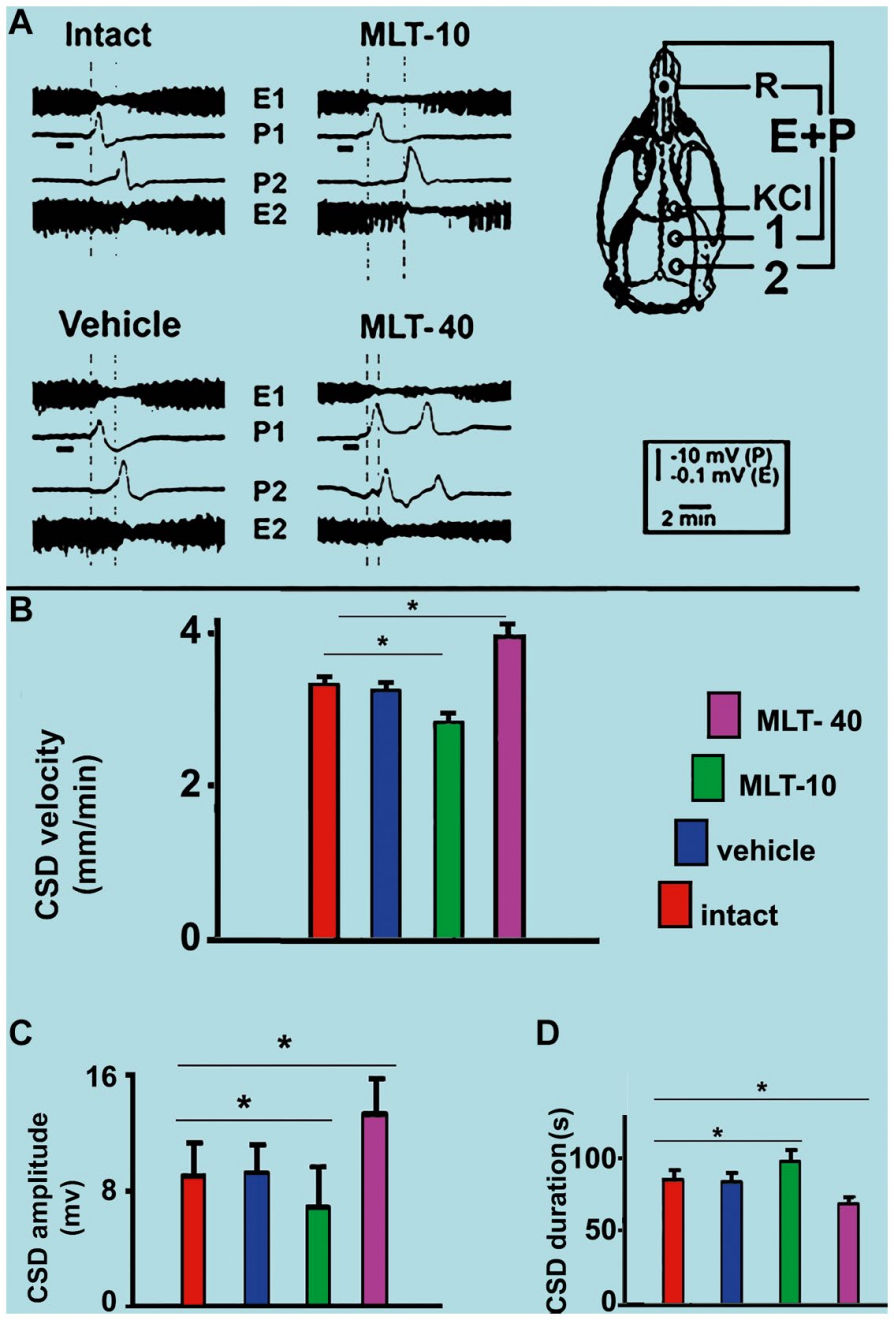
**(A)** Representative traces illustrating the typical ECoG depression (E) and the slow potential change (P) of cortical spreading depression (CSD). These traces were recorded at two cortical points (1 and 2) in two control (intact and vehicle) and two MLT-treated, 36–42-day-old male rats. The skull diagram shows the first and second recording positions (1 and 2), from which the traces marked with the same numbers were obtained. The position of the standard reference electrode (R) on the nasal bones and the application point of the CSD-eliciting stimulus on the frontal cortex is also shown. CSD was elicited in the frontal cortex by chemical stimulation (a 1–2 mm diameter cotton ball soaked with 2% KCl) applied for 1 min on the intact dura mater, as indicated by the horizontal bars under the traces at point 1. The vertical dashed lines indicate the latency for a CSD wave to cross the interelectrode distance. Longer and shorter latencies were observed in the MLT-10 and MLT-40 groups, respectively. Panels **(B–D)** show the CSD propagation velocity, amplitude, and duration, respectively. Data are the mean values of 10–12 CSD episodes per rat from 20 controls (10 intact and 10 saline-treated rats) and 20 MLT-treated rats (10 MLT-10 and 10 MLT-40 animals) recorded over a 4-h recording session. ^*^p < 0.005 (ANOVA followed by the Holm–Sidak test).

Application of a cotton ball (1–2 mm in diameter) soaked in 2% KCl solution (approximately 270 mM) for 1 min, at 20 min intervals, to a point in the frontal cortex, usually elicited a single wave of CSD (Figure 5A). The propagating CSD wave was recorded by the two more posterior electrodes on the stimulated hemisphere (see recording points 1 and 2 in the skull diagram in Figure 5A).

Regarding the propagation velocity of CSD (Figure 5B), ANOVA indicated intergroup differences [*F*(3, 36) = 85.130; *p* < 0.001]. *Post hoc* (Holm–Sidak) test comparisons showed that the low and the high doses of MLT (10 and 40 mg/kg, respectively) significantly decelerated and accelerated CSD (2.86 ± 0.14 mm/min and 3.96 ± 0.16 mm/min, respectively; *p* < 0.01), compared to the control groups (3.31 ± 0.10 mm/min for the intact control and 3.25 ± 0.11 mm/min for the vehicle-treated groups).

The amplitude and duration of the negative component of the slow potential change, which is the hallmark of CSD, are shown in Figures 5C,D, respectively. ANOVA indicated a main effect of treatment for amplitude [*F*(3, 36) = 9.520; *p* < 0.001] and duration [*F*(3, 36) = 29.767; *p* < 0.001]. The mean amplitudes in the two control groups were 8.9 ± 2.4 mV and 9.0 ± 1.7 mV, respectively, for the intact and vehicle-treated groups. For the groups treated with MLT (MLT-10 and MLT-40), the amplitude was 6.8 ± 1.2 mV and 13.2 ± 2.3 mV, respectively, indicating that the treatment with the low and high doses of MLT was associated with a significantly lower and higher amplitude in comparison to the control groups. The Holm–Sidak test indicated that the two groups that were treated with 10 and 40 mg/kg MLT had longer and shorter CSD durations, respectively (96.5 ± 9.1 s and 70.7 ± 4.0 s, respectively), than the two control groups (83.9 ± 5.4 s and 82.1 ± 4.0 s for the intact and vehicle groups, respectively).

### 3.5. MDA and SOD levels in the cerebral cortex

Table 1 presents the levels of MDA and SOD in the cerebral cortex of control-and MLT-treated rats. ANOVA indicated intergroup differences [*F*(3, 16) = 6.152; *p* = 0.006 for MDA, and 12.293; *p* = 0.001 for SOD]. The *post hoc* test indicated that the MLT-10 group, but not the MLT-40 group, presented with lower levels of MDA (3.34 ± 1.24 nMol/mg protein) and higher levels of SOD (19.49 ± 4.57 AU/min/mg protein), as compared to the intact (MDA, 6.21 ± 0.58 nMol/mg protein; SOD, 7.39 ± 3.88 AU/min/mg protein) and vehicle controls (MDA, 6.93 ± 2.53 nMol/mg protein; SOD, 7.59 ± 3.71 AU/min/mg protein) [Holm–Sidak *post hoc* test; *p* < 0.01].

**Table 1:** MDA and SOD levels in the cerebral cortex of 36–42-day-old rats (*n* = 20) previously treated (5 rats per group) every other day, from postnatal day 7 to 27, with 10 or 40 mg/kg/d melatonin (groups MLT-10 and MLT-40, respectively), or vehicle, or not treated at all (intact group).

Treatment group (*n* = 5 per group)	MDA level (nMol/mg protein)	SOD (AU/min/mg prot.)
Intact	6.21 ± 0.58	7.39 ± 3.88
Vehicle	6.93 ± 2.53	7.59 ± 3.71
MLT-10	3.34 ± 1.24^*^	19.49 ± 4.57^**^
MLT-40	4.32 ± 0.82	11.00 ± 3.33

Data are expressed as mean ± standard deviation. ^*^Significantly different from the intact and vehicle groups ^**^Significantly different from the other three groups (*p* < 0.01; ANOVA followed by the Holm–Sidak test).

## 4. Discussion

For the first time, we have documented, in the brains of developing rats, the differential, dose-dependent effects of MLT treatment on behavioral (anxiety and memory) and electrophysiological (spreading depression) aspects of brain function. Our data from the anxiety and memory tasks suggest an anxiolytic effect of the lower dose but not the higher dose of MLT (see Figures 3, 4). In addition, our CSD findings indicate that MLT may act differentially in the brain at low and high doses, decelerating and accelerating CSD propagation, respectively (see Figure 5).

The introduction mentions that MLT1 and MLT2 receptors are predominantly found in subcortical brain regions (Dubocovich et al., 1998; Lacoste et al., 2015). Therefore, these receptors are unlikely to directly mediate the MLT action on a cortically-based phenomenon like CSD. However, we cannot exclude the role of MLT receptors in processes such as anxiety and memory. On the other hand, MLT at physiological concentrations protects mitochondria from oxidative damage (Zhang and Zhang, 2014). *In vitro* evidence suggests that, depending on the concentration, MLT can generate functionally significant amounts of ROS (Zhang H. et al., 2011; Zhang H. M. et al., 2011; Martinis et al., 2012; Zhang and Zhang, 2014). As in other organs, the biochemical and morphological organization of the brain can be altered by oxidative stress after the imbalance represented by the increase in ROS production; on the other hand, the decrease in the effectiveness of antioxidant systems can also alter the redox imbalance (Guedes et al., 2012; Mendes-da-Silva et al., 2014). When this imbalance occurs early in life, the deleterious consequences for brain functioning may be more severe than in the adult brain (Grantham-Mcgregor, 1995; Guedes et al., 2012; Colella et al., 2016).

The absence of a weight gain difference after the administration of MLT (Figure 2A) is in line with the fact that the animals in the four groups of this study were subjected to the same conditions of food ingestion (free access to food) and caloric expenditure. Food intake and caloric expenditure were not presently calculated; notwithstanding that, the similar weight gain in all groups suggests no relevant effect of MLT on those parameters under the experimental conditions and age range of the animals in our experiment. The young animals from this study were clinically healthy; no significant difference in blood glucose levels was found between the MLT-treated and control groups (Figure 2B). Our speculations in this regard are limited, as we did not measure insulin and lipid levels that could prove any relationship with MLT. However, some studies reported a greater insulin secretion after MLT administration, linking oscillations in the circadian rhythm to diabetes development [see the recent study by Xia et al. (2023)].

The interpretation of the parameters provided by the behavioral tests with rodents is based on established behavioral neuroscience literature (see Améndola et al., 2022). The present results revealed that MLT had an anxiolytic action (Figure 3) and positively impacted object recognition memory tests (Figure 4). In the OF task, the number of entries into the center and the time spent in this area represent a “risky activity” for the animal. Therefore, the MLT-10 group exhibited anxiolytic-like behavior compared to all other groups. In the EPM task, the “risky activity” is represented by the number of entries into the open arms and the time spent in these areas (Pellow et al., 1985). The animals that received the lower dose of MLT (10 mg/kg) entered more frequently and remained longer in the aversive region (open arms). Therefore, the MLT-10 group, but not the MLT-40 group, showed an anxiolytic-like behavior compared to the Control and Intact groups.

In the PR and SR tasks, the discrimination index calculates cognitive performance—represented by sensory-spatial ability—(see section 2). The higher the index, the better the animal’s cognitive performance. Therefore, the MLT-10 group showed better cognitive activity in the spatial recognition task than all other groups. The results of OF and EPM indicate that the MLT-treated rats presented less anxiety behavior and improved memory, and surprisingly, these effects were significant at the low MLT dose. We suggest this is related to the dose-associated redox imbalance, as our brain MDA and SOD measurements indicated (Table 1). Indeed, the decreased levels of MDA and increased levels of SOD in the MLT-10 group suggest that melatonin at this dose had an antioxidant effect in the developing brain. A recent study (Al Gburi et al., 2023) came to the same suggestion.

Childhood and adolescence are crucial for developing learning, memory, and emotional responses (Sun et al., 2021). MLT has anti-inflammatory, anti-amyloidogenic, and antioxidant properties that underlie its potential to reduce brain damage resulting from inflammatory, amyloidogenic, and oxidant processes and improve learning and memory deficits with long-term treatment (Zakaria et al., 2016; Iwashita et al., 2021). A single administration of MLT facilitates cognitive performance in humans (Foster et al., 2014).

This study used electrophysiological analysis of CSD parameters to identify MLT’s possible antioxidant (CSD deceleration) and prooxidant (CSD acceleration) actions. Some exogenous and endogenous antioxidant compounds can, under certain circumstances, exert prooxidant activities. That is the case for tocopherol (Gogvadze et al., 2010) and ascorbate (Otero et al., 1997; Podmore et al., 1998; Doseděl et al., 2021). Our group has demonstrated that rats treated with increasing doses of ascorbic acid displayed a biphasic effect of this treatment on the propagation of CSD, with a deceleration of the phenomenon at a lower dose and an acceleration of CSD with higher doses. The CSD decelerating effect of low doses of ascorbic acid was associated with decreased malondialdehyde levels in the brain, compared with those in the corresponding saline and intact groups (Mendes-da-Silva et al., 2014).

The mechanisms by which high concentrations of MLT could stimulate the production of ROS have yet to be determined. Zhang and Zhang (2014) suggested that the weak interaction between calmodulin and MLT might be involved in the stimulation of ROS production. One possible speculation is that different concentrations of MLT may differentially modulate the subcellular localization of calmodulin, thus dictating its involvement in prooxidant vs. antioxidant activities. Calmodulin is a protein that binds with low affinity to MLT and appears to mediate MLT prooxidant action. Furthermore, MLT can interact with mitochondria to promote the generation of ROS. Notably, applying a high concentration of KCl to the rat cortex (a procedure that indeed triggers CSD) influences the expression of calmodulin (Torrão et al., 2002).

*In vivo* evidence indicates that MLT is a potent antioxidant at typical pharmacological concentrations but may have a prooxidant action in the presence of copper ions (Wang et al., 2019b). We do not know whether brain copper levels were altered in our animals; probably not. However, it is worth mentioning that, under this curious condition, melatonin can act as a prooxidant molecule (Wang et al., 2019a,b). In *in vitro* experiments, the prooxidant action of MLT promotes inflammatory responses and apoptosis (Wolfler et al., 2001; Radogna et al., 2010). Interestingly, the extracellular increase in ROS facilitates CSD elicitation and propagation, both *in vitro* (Netto and Martins-Ferreira, 1989) and *in vivo* (El-Bachá et al., 1998). CSD is modulated by changes in brain excitability (Koroleva and Bures, 1980; Guedes and Abadie-Guedes, 2019) and is influenced by the production of ROS-like superoxide anions in brain tissue (El-Bachá et al., 1998; Sousa et al., 2021). Although we did not measure ROS directly, we measured MDA and SOD levels in the cerebral cortex. These molecules are cellular markers of redox imbalance because they change proportionally to the amount of ROS. MLT can also exert anti-excitotoxic effects through its neuroprotective action involving gamma-aminobutyric acid (GABA) as a mediator (Pandi-Perumal et al., 2013). This possibility is supported by studies indicating that MLT protects neurons from the toxicity of beta-amyloid peptide (the main neurotoxin involved in Alzheimer’s disease) by activating GABAergic receptors (Louzada et al., 2004). After hypoxia injury in rats, MLT administration reduced glutamate levels and hypoxia-induced structural damage to neurons, axons, and dendrites in the brainstem, suggesting that it can ameliorate excitotoxic injury (Kaur et al., 2011). Notably, gamma-aminobutyric acid and glutamate neurotransmitters are causally related to the neuroexcitability influences on CSD (Lima et al., 2009; Barhorst et al., 2022).

The findings of this study cannot be attributed to the use of negligible amounts of ethanol to dissolve MLT or to the stress related to intraperitoneal injection, as the two control groups (vehicle and intact animals) showed similar behavioral reactions and CSD features. The oral route of administration is clinically the most used in the case of MLT. When ingested orally, only 15% of the MLT dose reaches the systemic circulation; the remaining 85% may undergo significant first-pass metabolism (Demuro et al., 2000). In addition, MLT does not appear to produce physiological or psychological dependence (Andersen et al., 2016).

## 5. Limitations of this study

### 5.1. Animal’s sex

Although we recognize sex as an essential biological variable on the effects of endogenous melatonin (Kawabata-Sakata et al., 2020), our study included only male rats. This limitation occurred because the hormonal oscillations of the female rat due to the estrous cycle are fast and complex and can alter the parameters that we addressed currently, including CSD (Accioly and Guedes, 2020). The need to use males only becomes even more evident when we use rats in the age of sexual maturation (i.e., from PND 35; Sengupta, 2013). Therefore, in various study groups in fundamental neuroscience, it is common to invest an initial effort in male rats and only then, with a well-established database, apply the same methodologies in female rats.

### 5.2. Endogenous MLT

Even though we evaluated the administration of exogenous MLT at two concentrations (10 and 40 mg/kg), we did not measure the impact of endogenous MLT—and its physiological oscillations—on electrophysiological parameters, for example. In this study, all experiments were performed during the dark phase of the circadian cycle, when the concentration of endogenous MLT is known to be higher (Govindarajulu et al., 2022). However, it would be interesting to study a control group in which the experiments were carried out in the rat’s light phase.

### 5.3. Routes of exogenous MLT administration

According to Yeleswaram et al. (1997), after intraperitoneal administration of 10 mg/kg Melatonin in rats, only 74% of this initial amount is bioavailable in the plasma due to the hepatic first-pass metabolism. Such pharmacokinetic effect is also observed in the oral route (Demuro et al., 2000). So, to evaluate the importance of the initial metabolism of the drug, it would be interesting to compare it with another parenteral route (e.g., intradermal, intravenous, or intracerebroventricular).

### 5.4. MLT-receptor dependent vs. independent action

Although we evaluated some of the receptor-independent effects of MLT—e.g., oxidative stress markers—we did not analyze in-depth MLT receptor expression or their downstream impacts. As stated in the Discussion, it is unlikely that these receptors mediate the MLT action on a cortically-based phenomenon like CSD, but we cannot exclude the role of MLT receptors in processes such as anxiety and memory (Dubocovich et al., 1998; Lacoste et al., 2015). Therefore, we encourage future work on this topic.

In conclusion, our findings demonstrate that the behavioral and electrophysiological effects of MLT treatment early in life differentially change as a function of the MLT dose. In behavioral parameters suggestive of anxiety and memory, MLT diminished anxiety and improved object recognition memory at the low dose but not at the high dose. Accordingly, MLT decelerated CSD propagation at the low dose (10 mg/kg) and accelerated CSD at the high dose (40 mg/kg) on the developing rat cortex. In this study, MLT at the low dose (but not at the high dose) was associated with significantly lower MDA and higher SOD cortical levels than the control groups. Based on these behavioral, electrophysiological, and biochemical pieces of evidence, we suggest that, in addition to the receptor-mediated MLT actions (Dubocovich et al., 1998; Lacoste et al., 2015), MLT acts as an antioxidant when given in low doses and may act as a prooxidant when given in high doses, as occurred with CSD under treatment with ascorbic acid (Mendes-da-Silva et al., 2014). However, confirming this suggestion requires a more detailed evaluation of the redox imbalance in the brain.

It is still uncertain whether this new effect of MLT can be extrapolated from the rat to the developing human brain (Vine et al., 2022; Reiter et al., 2023). However, the clinical evidence suggests that melatonin may improve sleep disorders, cognitive functioning (Sumsuzzman et al., 2021), and neonatal hypoxic–ischemic brain lesions (Cardinali, 2019). In addition, melatonin may benefit classical migraine patients (Ebert et al., 1999) and improve blood glucose levels in diabetes (Delpino et al., 2021). Interestingly, melatonin levels are reduced in some metabolic, cardiovascular, and neurological diseases (Cardinali, 2020). This evidence suggests a possible relevant implication of our novel findings in the developing rat brain.

## Data availability statement

The raw data supporting the conclusions of this article will be made available by the authors, without undue reservation.

## Ethics statement

The animal study was approved by CEUA—Comitte de Etica e Uso de Animais da UFPE (approval protocol no. 0006/2020). The study was conducted in accordance with the local legislation and institutional requirements.

## Author contributions

AA: Data curation, Formal analysis, Investigation, Methodology, Project administration, Visualization, Writing – original draft. MF-d-O: Conceptualization, Formal analysis, Investigation, Validation, Writing – review & editing. AN: Conceptualization, Formal analysis, Methodology, Writing – review & editing. VO: Data curation, Methodology, Writing – review & editing. JC: Conceptualization, Formal analysis, Investigation, Methodology, Writing – original draft. LV: Conceptualization, Formal analysis, Methodology, Writing – review & editing. RG: Conceptualization, Formal analysis, Funding acquisition, Supervision, Writing – review & editing.
